# Associations of eHealth literacy and dietary diversity with depressive symptoms among patients with chronic diseases in rural Western China

**DOI:** 10.3389/fdgth.2026.1920279

**Published:** 2026-09-25

**Authors:** Yanan Chen, Ting Zhao, Chunsheng Li, Haodong Meng, Hui Qiao

**Affiliations:** 1 School of Public Health, Ningxia Medical University, Yinchuan, China; 2 Key Laboratory of Environmental Factors and Chronic Disease Control, Ningxia Medical University, Yinchuan, China

**Keywords:** depressive symptoms, dietary diversity, eHealth literacy, latent profile analysis, patients with chronic disease

## Abstract

**Objective:**

The global prevalence of depressive symptoms has been increasing steadily. Both eHealth literacy and dietary diversity are closely linked to mental health. However, the mechanisms by which these factors jointly influence depressive symptoms remain unclear. This study aimed to investigate whether dietary diversity mediates the association between eHealth literacy and depressive symptoms among patients with chronic diseases in rural western China while also evaluating the interaction and combined effects of these two factors.

**Methods:**

This cross-sectional study utilized data from the 2024 Ningxia Rural Residents’ Health Status and Health Service Utilization Survey. The final analysis included 2,655 patients aged 18 years and older with chronic diseases. Latent profile analysis (LPA) was employed to categorize eHealth literacy levels. A mediation analysis was performed to investigate the mediating role of dietary diversity in the relationship between eHealth literacy and depressive symptoms. To evaluate the interactive and joint effects of eHealth literacy and dietary diversity on depressive symptoms, multiplicative interaction models, additive interaction models, and multivariable logistic regression models were applied.

**Results:**

Using LPA, participants were classified into three groups on the basis of eHealth literacy: high (34.43%), medium (19.43%), and low (46.14%). Low eHealth literacy was linked to increased odds of depressive symptoms (OR = 1.64, 95% CI: 1.49 to 1.82), a relationship that diminished after adjusting for dietary diversity (OR = 1.53, 95% CI: 1.38 to 1.72). Dietary diversity served as a partial mediator in this association, explaining 18.79% of the total effect. A significant additive interaction was identified (RERI = 0.65; 95% CI: 0.12 to 1.19), whereas the results of the joint analysis revealed the lowest odds of depressive symptoms in the high-literacy/high-dietary-diversity group (OR = 0.17; 95% CI: 0.05 to 0.56).

**Conclusions:**

Dietary diversity may partially mediate the association between eHealth literacy and depressive symptoms. A synergistic association exists between eHealth literacy and dietary diversity with respect to depressive symptoms. Within various eHealth literacy subgroups, dietary diversity is correlated with reduced odds of depressive symptoms, underscoring its potential significance for promoting mental health.

## 1 Introduction

Mental health represents a significant public health concern with far-reaching implications for social and economic development. The World Health Organization estimates that approximately 5.7% of adults worldwide experience depression (1), which is anticipated to become the leading cause of disability globally by 2030 (2). In China, depressive disorders are the second leading cause of years lived with disability (3), with a lifetime prevalence of 6.8% and a 12-month prevalence of 3.6%, impacting more than 95 million individuals (4). Additionally, a recent secondary analysis of the China Mental Health Survey (CMHS) focusing on older adults with noncommunicable diseases (NCDs) revealed an even greater burden, with a lifetime prevalence of 10.1% and a 12-month incidence of 5.4% (5).

Chronic diseases represent a significant burden on human health. More than 43 million individuals worldwide die annually from chronic diseases, which account for approximately three-quarters of all global deaths (6). The burden of chronic conditions exceeds half of the total global disease burden (7), posing a substantial public health challenge that impacts economic and social development. Long-term illnesses and chronic pain can diminish the quality of life for patients with chronic diseases, potentially leading to poor mental health and depression (8). Research has indicated that the prevalence of depression among patients with chronic diseases in China is substantial and increasing. A 2025 study examining the Chinese population with noncommunicable chronic diseases revealed a depression prevalence of 32.62% (9). Additionally, a separate 2025 analysis involving 9,535 Chinese participants aged 45 and older revealed that the detection rate of depressive symptoms was 29.6% among those with multiple chronic conditions (10). Furthermore, depression and anxiety have emerged as independent risk factors for increased mortality among patients with chronic diseases (11).

The internet is increasingly recognized as a vital channel through which the public can access health information and services (12–14). The ability to seek, locate, comprehend, and evaluate health information from electronic sources, as well as to apply the acquired knowledge to address or resolve health issues, is termed eHealth literacy (15). Research has indicated that individuals’ eHealth literacy is closely linked to their effectiveness in utilizing internet-based health information (16). Participants with higher eHealth literacy are more inclined to search for health information, engage in health-related behaviors, and practice health self-management, which enhances their understanding of their health status and contributes to better overall health (17, 18). However, individuals in rural areas encounter multiple disadvantages related to economic development, social security, and health conditions. Their limited access to essential resources, such as quality healthcare and health information, significantly hampers their ability to prevent and cope with disease risk. Studies have demonstrated that the overall level of eHealth literacy among rural people is generally low (19, 20), and individuals with lower eHealth literacy face a markedly increased risk of depressive symptoms (21).

A growing body of research has identified dietary factors, including overall dietary levels and specific dietary patterns, as significant correlates of depression (22). Dietary diversity, defined as the consumption of a variety of foods across and within food groups to achieve comprehensive nutrition, serves as a key indicator of dietary quality. Accumulating evidence indicates that increased dietary diversity not only helps prevent malnutrition and reduce the risk of chronic diseases but also may foster positive emotions and enhance psychological well-being through multiple pathways (23–25). Similarly, a cross-sectional study involving older adults in China revealed that greater dietary diversity was significantly associated with a reduced risk of depression (26). Furthermore, a recent systematic review and meta-analysis confirmed a significant inverse relationship between dietary diversity and depressive symptoms (27, 28).

Emerging evidence indicates a potential link between eHealth literacy and dietary behaviors, which is particularly relevant to the present study. Individuals with higher eHealth literacy are more inclined to seek, comprehend, and apply health information to enhance their dietary practices (29, 30). However, whether dietary diversity acts as a mediating pathway in the relationship between eHealth literacy and depressive symptoms remains unclear. In addition to this potential mediation, eHealth literacy and dietary diversity may interact with one another concerning depressive symptoms. Nonetheless, the interaction and joint effects of these two factors on depressive symptoms have received limited empirical investigation.

Given the dual burden of a high incidence of chronic diseases and low health literacy in rural China, understanding how eHealth literacy is associated with depressive symptoms through dietary diversity, as well as how these two factors collectively influence depressive symptoms, is crucial. This understanding is vital for developing effective intervention strategies. However, previous studies have primarily examined these factors in isolation, and systematic investigations of their mediating and joint effects remain inadequate. To address these gaps, this study focused on patients with chronic diseases in rural western China, with the following objectives: (1) to explore the pathways through which eHealth literacy and dietary diversity are related to depressive symptoms while testing the mediating role of dietary diversity in this relationship; and (2) to assess the interaction and joint effects of eHealth literacy and dietary diversity on depressive symptoms. This research aims to provide scientific evidence for the development of comprehensive chronic disease management strategies that integrate digital health promotion and nutritional intervention.

## 2 Methods and measurement

### 2.1 Data sources and sampling methods

This study employed a cross-sectional design. Data were obtained from the 2024 survey on health status and health service utilization among rural residents in Ningxia. This survey was conducted within a natural population cohort established in 2009, which has been followed up in six waves over 15 years (2011, 2012, 2015, 2019, 2022, and 2024). The respondents included rural individuals aged 18 years and older from Haiyuan, Yanchi, Pengyang, and Xiji Counties in Ningxia. Informed consent was obtained from all the respondents prior to the study. This research adhered to the principles of the Helsinki Declaration and received approval from the Institutional Review Board of Ningxia Medical University (2023-G157).

The questionnaire utilized in this study was developed by experts and scholars from Ningxia Medical University in collaboration with the project team. Prior to conducting door-to-door household surveys, surveyors underwent uniform training. Each household completed a single form, with surveyors administering questions face-to-face in accordance with the questionnaire, which addressed topics such as basic personal information, illnesses and medical treatments within the past two weeks, hospitalizations over the past year, chronic disease prevalence, health status, eHealth literacy, and healthy lifestyles of individuals aged 18 and older. During the design phase, we refined and optimized the questionnaire content through consultations with experts. Before the formal investigation, we conducted a preliminary survey to identify existing issues and made necessary adjustments. This series of revisions significantly enhanced the quality of the questionnaire. To ensure scientific rigor and regional representativeness, we employed a multistage stratified sampling method, initially randomly selecting four representative counties (Haiyuan, Yanchi, Xiji, and Pengyang) in the southern mountainous region of Ningxia. All administrative villages within these counties were subsequently classified according to socioeconomic indicators (high, medium, and low). Within each stratum, 40% of the natural villages were randomly selected as sample villages. Using systematic sampling, we selected 20 to 33 households from the roster of each sample village.

We selected patients with chronic diseases who had resided in the area for more than 6 months and who were 18 years of age or older as research subjects. In this study, chronic diseases were defined as any physician-diagnosed condition reported by participants within the past year. The sample size was determined on the basis of the anticipated prevalence of depressive symptoms (32.62%) among patients with chronic diseases (9), resulting in a required sample size of 830 participants (*α* = 0.05, margin of erro*r* = 10%). After accounting for a 20% nonresponse rate, the target sample size was established at 1,038 participants. The final analysis included 2,655 participants, significantly exceeding the required sample size.

To ensure accuracy, we performed logical error corrections on the database and eliminated samples with substantial missing or ambiguous variable data. Specifically, participants with incomplete data for any key variable, such as the Patient Health Questionnaire-9 (PHQ-9), the eHealth Literacy Scale (eHEALS), the Dietary Diversity Score (DDS), or covariates, were excluded through complete-case analysis. No imputation was conducted. Ultimately, 2,655 participants were included in the final analysis and categorized into two groups on the basis of the PHQ-9: the nonsignificant depressive symptoms group and the depressive symptoms group. The detailed selection process is depicted in Figure 1.

**Figure 1:**
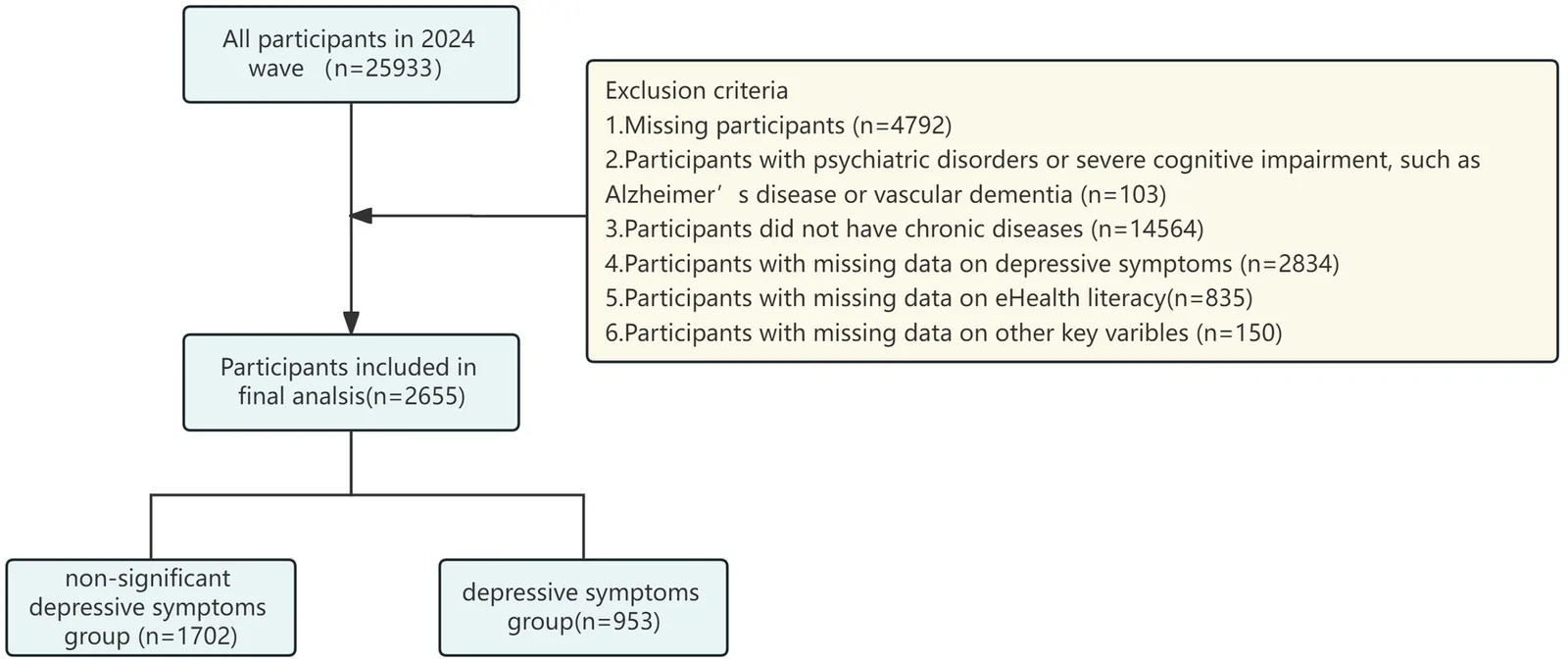
Flow diagram of the inclusion and exclusion criteria.

### 2.2 Measurement

#### 2.2.1 eHealth literacy

eHealth literacy was evaluated using the Chinese version of the eHealth Literacy Scale (eHEALS), which has demonstrated strong reliability and validity in previous research. The validation study reported a Cronbach's *α* of 0.94 and an intraclass correlation coefficient (ICC) of 0.92 for test-retest reliability. Additionally, construct validity was supported by anticipated correlations with health literacy, self-assessed internet skills, and the use of the internet for health information (31, 32). The scale consists of eight items across three dimensions: the ability to apply online health information and services, the ability to evaluate online health information, and the ability to make decisions on the basis of online health information. Each item is rated on a 5-point Likert scale, ranging from “1 = strongly disagree” to “5 = strongly agree.” The total score, which is the sum of all item scores, ranges from 8 to 40, with higher scores indicating greater levels of eHealth literacy. In this study, the Cronbach's *α* coefficient for eHealth literacy was 0.983.

#### 2.2.2 Depressive symptoms

The PHQ-9 is a widely utilized tool for screening depression in NHANES and is commonly applied in primary care settings (33). It evaluates the severity of depressive symptoms experienced by respondents over the prior two weeks (34). The scale consists of nine items, each rated on a 0–3 scale: “Not at all = 0,” “Several days = 1,” “More than half the days = 2,” and “Nearly every day = 3.” The total score can range from 0 to 27 points. In accordance with the original PHQ-9 guidelines, a score of ≥5 signifies mild or higher depressive symptoms and is the recommended screening threshold in primary care and community environments (33). Consequently, a cutoff of ≥5 was employed in this study to define the presence of depressive symptoms. To offer a comprehensive overview of the symptom severity spectrum, the distribution of participants under the stricter cutoff of ≥10 points, which indicates moderate-to-severe depressive symptoms, is also presented in Supplementary Table S1. A higher score indicates more severe depressive symptoms. In this study, the Cronbach's *α* coefficient for the PHQ-9 was 0.898.

#### 2.2.3 Dietary diversity

Dietary diversity was evaluated using the Dietary Diversity Score (DDS), which is based on the nine food groups outlined in the 2016 edition of the Chinese Resident's Balanced Dietary Pagoda: (1) grains and tubers; (2) vegetables; (3) fruits; (4) fish and shellfish; (5) livestock and meat; (6) eggs; (7) dairy and dairy products; (8) legumes; and (9) oils. Foods outside these categories, including nutritional supplements, carbonated beverages, and candies, were excluded from the assessment. The respondents reported their dietary intake over the preceding three days. A score of 1 was assigned for each of the nine food groups if the respondent consumed any food from that group during the recall period; a score of 0 was assigned otherwise. Consumption of multiple items within the same group was counted only once, and neither the frequency nor the quantity of intake was considered. The total DDS score, which ranged from 0 to 9, was calculated as the sum of all item scores, with higher scores indicating greater dietary diversity (35). On the basis of the total score, dietary diversity was categorized into three levels: inadequate (<4 points), moderate (4–6 points), and adequate (≥7 points) (36).

### 2.3 Statistical analysis

The baseline characteristics of the participants were delineated according to varying levels of eHealth literacy. Normally distributed continuous variables are reported as the mean ± standard deviation (SD), whereas skewed continuous variables are presented as the median and interquartile range (IQR). Categorical variables are expressed as frequencies and percentages. Data were summarized on the basis of eHealth literacy categories and compared using analysis of variance (ANOVA), chi-square tests, or the Kruskal‒Wallis test, as appropriate. To classify eHealth literacy patterns, this study utilized latent profile analysis (LPA) (37, 38). This choice was informed by three key considerations. First, eHealth literacy is a multidimensional construct that includes functional, interactive, and critical skills; LPA effectively captures the combined patterns of these dimensions at the individual level, whereas a single total score obscures the heterogeneity in skill configurations. Second, traditional categorization methods depend on arbitrary cut-offs and assume population homogeneity, which may not accurately reflect the actual distribution of eHealth literacy in rural samples. Third, as a person-centered approach, LPA identifies subgroups with similar response profiles, rendering it particularly appropriate for heterogeneous populations, such as rural patients with chronic diseases, where varying skill combinations may not align with a simple linear gradient.

Potential covariates, including age, sex, marital status, educational level, occupation, self-rated health status, annual income, smoking status, alcohol consumption status, sleep duration, and exercise frequency, were identified as potential confounders on the basis of the literature (39, 40). T These variables underwent systematic screening through univariate analyses, retaining only those with *P* < 0.05. Following this screening, age, sex, educational level, self-rated health status, occupation, alcohol consumption status, and sleep duration emerged as the final covariates, which were consistently adjusted for in all subsequent statistical models (Supplementary Table S2).

Spearman rank correlation analysis was employed to investigate the relationships among eHealth literacy, dietary diversity, and depressive symptoms. All three variables were treated as continuous, utilizing the total scores of the eHEALS (range: 8–40), DDS (range: 0–9), and PHQ-9 (range: 0–27). A mediation analysis was conducted to determine whether dietary diversity mediated the association between eHealth literacy and depressive symptoms, following the counterfactual framework proposed by Imai (41). The model estimated (1) the indirect effect (ACME) of eHealth literacy on depressive symptoms via dietary diversity; (2) the direct effect (ADE) that was not mediated by dietary diversity; and (3) the proportion of the effect that was mediated. Bootstrapping with 1,000 resamples was used to derive 95% confidence intervals for the mediating effect. To evaluate the robustness of the regression models, multicollinearity was assessed using the variance inflation factor (VIF), and the overall model fit was analyzed using Nagelkerke's R^2^.

Multivariate logistic regression models were employed to estimate the odds ratios (ORs) and 95% confidence intervals (CIs) for the associations between eHealth literacy, dietary diversity, and depressive symptoms. Model 1 incorporated eHealth literacy groups as the independent variable while adjusting for the aforementioned covariates. Model 2 further included the Dietary Diversity Score (DDS). The differences in ORs between Model 1 and Model 2 were analyzed to assess the explanatory role of dietary diversity.

Further stratified analyses were conducted on the basis of the latent classes of eHealth literacy to investigate the association between dietary diversity and depressive symptoms within each eHealth literacy subgroup. In these analyses, individuals exhibiting insufficient dietary diversity (DDS 0–3) were designated as the reference group to assess whether a diverse diet provided a protective effect against depressive symptoms across various eHealth literacy subgroups. To evaluate the multiplicative and additive interactions between eHealth literacy and dietary diversity, these variables were dichotomized into extreme groups (low vs. high eHealth literacy; DDS 0–3 vs. 7–9) to increase statistical power and mitigate the risk of multiple comparisons. This 2 × 2 interaction analysis was employed to calculate the product term (representing the multiplicative interaction) and the relative excess risk due to interaction (RERI). The ORs and 95% CI for the product term serve as indicators of multiplicative interaction, whereas the RERI and its 95% CI function as measures of additive interaction.

To evaluate joint associations, participants were categorized into nine groups based on three levels of eHealth literacy (low, medium, and high) and three levels of DDS (0–3, 4–6, and 7–9). The group with low eHealth literacy and a DDS of 0–3 served as the reference group, and the ORs for depressive symptoms were assessed across the remaining eight groups to determine the dose‒response relationship. To ensure the robustness of our findings, two sets of sensitivity analyses were conducted: one that treated the PHQ-9 score as a continuous variable and the other that applied a stricter cutoff of ≥10 points. Both analyses involved rerunning the multivariable regression models and mediation analyses. Missing data were addressed through complete-case analysis (listwise deletion). All the statistical analyses were executed using Mplus 8.3 and R 4.4.2, with a two-sided *P* value of <0.05 deemed to indicate statistical significance.

## 3 Results

### 3.1 Latent profile analysis of eHealth literacy

Latent profile analysis was performed to investigate the underlying profiles of eHealth literacy. As indicated in Supplementary Table S3, although the AIC, BIC, and aBIC values consistently decreased, only the 2-class and 3-class models demonstrated significant improvements in fit, as evidenced by the BLRT and VLMR results (*P* < 0.01). Given the high classification accuracy (entropy = 0.979) and the nonsignificant LRT outcomes for the 4-class and 5-class models, the 3-class solution was selected as the optimal model.

The profile plot of the 3-class model is shown in Supplementary Figure S1. On the basis of the mean scores across the eight eHealth literacy items, these three profiles were designated the low, medium, and high eHealth literacy groups. The profiles exhibited parallel, noncrossing trajectories across the items, reflecting distinct levels of overall eHealth literacy and establishing a natural gradient from low to high proficiency. This classification facilitated the identification of specific vulnerable subgroups, notably 46.14% in the low group, within the rural chronic disease population.

### 3.2 Population characteristics

Of the 25,933 participants initially screened, 23,278 were excluded, including 3,819 because of missing data on key variables. To evaluate selection bias, we compared the baseline characteristics of the excluded participants (*n* = 3,819) with those included in the analysis (*n* = 2,655). No significant differences were noted in key measures such as age, dietary diversity, or depressive symptoms; however, differences were identified in sex, smoking status, or drinking behavior (Supplementary Table S4). Among the 2,655 participants (mean age 63.04 years; 57.9% men), 914 (34.43%) were classified as having high eHealth literacy, 516 (19.43%) as having medium eHealth literacy, and 1,225 (46.14%) as having low eHealth literacy. Significant differences were observed across the three groups concerning age, marital status, education level, occupation, self-rated health status, depressive symptoms, dietary diversity, smoking status, alcohol consumption, and income (*P* < 0.05). Specifically, individuals with low eHealth literacy were older and less educated. The prevalence of depressive symptoms in the overall population was 35.89%, with a significantly greater prevalence in the low eHealth literacy group (42.12%) than in the high eHealth literacy group (29.43%). Furthermore, the mean dietary diversity score decreased as eHealth literacy levels decreased (7.01 ± 1.40 in the high group vs. 6.58 ± 1.53 in the low group). Detailed information on the baseline characteristics is provided in Table 1.

**Table 1:** Baseline characteristics stratified by eHealth literacy in patients with chronic diseases.

Characteristics	Total population (*n* = 2655)	High eHealth Literacy (*n* = 914)	Medium eHealth Literacy (*n* = 516)	Low eHealth Literacy (*n* = 1225)	*P*
**Age (years), mea*n* ± SD**	63.04 ± 11.03	59.76 ± 11.69	61.32 ± 10.12	66.21 ± 9.96	< 0.001
**Gender, *n* (%)**					0.590
Men	1538 (57.93)	540 (59.08)	301 (58.33)	697 (56.90)	
Women	1117 (42.07)	374 (40.92)	215 (41.67)	528 (43.10)	
**Marital status, *n* (%)**					0.027
Married	2607 (98.19)	897 (98.14)	500 (96.90)	1210 (98.78)	
Unmarried	48 (1.81)	17 (1.86)	16 (3.10)	15 (1.22)	
**Educational level, *n* (%)**					< 0.001
Uneducated	1116 (42.03)	288 (31.51)	190 (36.82)	638 (52.08)	
Primary school	986 (37.14)	334 (36.54)	218 (42.25)	434 (35.43)	
Junior high school and above	553 (20.83)	292 (31.95)	108 (20.93)	153 (12.49)	
**Occupation, *n* (%)**					0.001
Agricultural work	1971 (74.24)	654 (71.55)	414 (80.23)	903 (73.71)	
Non-Agricultural work	684 (25.76)	260 (28.45)	102 (19.77)	322 (26.29)	
**Self-rated health status, *n* (%)**					< 0.001
Very good	44 (1.66)	17 (1.86)	10 (1.94)	17 (1.39)	
Good	288 (10.85)	130 (14.22)	59 (11.43)	99 (8.08)	
Fair	645 (24.29)	243 (26.59)	143 (27.71)	259 (21.14)	
Poor	1073 (40.41)	394 (43.11)	195 (37.79)	484 (39.51)	
Very poor	605 (22.79)	130 (14.22)	109 (21.12)	366 (29.88)	
**Depressive symptoms, *n* (%)**					< 0.001
Yes	953 (35.89)	269 (29.43)	168 (32.56)	516 (42.12)	
No	1702 (64.11)	645 (70.57)	348 (67.44)	709 (57.88)	
**Dietary diversity, mean ± SD**	6.79 ± 1.49	7.01 ± 1.40	6.95 ± 1.45	6.58 ± 1.53	0.039
Smoking status, ***n* (%)**					0.004
Current smoke	577 (21.73)	232 (25.38)	104 (20.16)	241 (19.67)	
Never smoker	2078 (78.27)	682 (74.62)	412 (79.84)	984 (80.33)	
**Alcohol consumption status, *n* (%)**					< 0.001
Current drinker	183 (6.89)	87 (9.52)	34 (6.59)	62 (5.06)	
Never drinker	2472 (93.11)	827 (90.48)	482 (93.41)	1163 (94.94)	
**Sleep duration, mean ± SD**	6.69 ± 1.58	6.84 ± 1.55	6.65 ± 1.57	6.59 ± 1.60	0.055
**Exercise frequency per week, *n* (%)**					0.180
0 times	1256 (47.31)	411 (44.96)	235 (45.55)	610 (49.80)	
1–2 times	312 (11.75)	106 (11.60)	64 (12.40)	142 (11.59)	
3–5 times	408 (15.37)	159 (17.40)	87 (16.86)	162 (13.22)	
≥6 times	679 (25.57)	238 (26.04)	130 (25.19)	311 (25.39)	
**Annual income, M (IQR)**	31271.00 (17460.00,56801.00)	11681.63(7064.00,18980.2)	11324.58(6730.31,18043.13)	9600.50(6399.00,16250.50)	<0.001

SD, standard deviation; IQR, interquartile range.

### 3.3 Common method bias test

In this study, Harman's single-factor test was utilized, and all the items pertaining to eHealth literacy, depressive symptoms, and dietary diversity were subjected to unrotated principal component factor analysis. The findings revealed eight factors whose eigenvalues exceeded 1. The first factor accounted for 25.34% of the variance, which falls short of the critical threshold of 40%, suggesting the absence of significant common methodological bias in this research.

### 3.4 Correlation analysis of eHealth literacy, dietary diversity and depressive symptoms

Table 2 presents the results of the correlation analysis of eHealth literacy, dietary diversity, and depressive symptoms in patients with chronic diseases. Spearman correlation analysis revealed a negative correlation between depressive symptoms and eHealth literacy (*ρ* = −0.119; *P* < 0.001) and between depressive symptoms and dietary diversity (*ρ* = −0.125; *P* < 0.001). In contrast, dietary diversity was positively correlated with eHealth literacy (*ρ* = 0.126, *P* < 0.001).

**Table 2:** Correlation analysis of eHealth literacy, dietary diversity and depressive symptoms in patients with chronic diseases.

Variable	eHealth literacy	Dietary diversity	Depressive symptoms
eHealth literacy	1	-	-
Dietary diversity	0.126**	1	-
Depressive symptoms	−0.119**	−0.125**	1

** *P* < 0.001.

### 3.5 Mediation effect of dietary diversity on the associations between eHealth literacy and depressive symptoms

On the basis of the findings from the descriptive and correlation analyses, a mediation model was constructed to examine dietary diversity as a mediator of the association between eHealth literacy and depressive symptoms. In this model, eHealth literacy served as the independent variable, depressive symptoms served as the dependent variable, and dietary diversity served as the mediating variable. Bootstrap analysis revealed a significant total effect of eHealth literacy on depressive symptoms (total effect = −0.031, 95% CI: −0.053 to −0.009). The direct effect of eHealth literacy on depressive symptoms remained significant after dietary diversity was incorporated (ADE = −0.026, 95% CI: −0.046 to −0.004). Additionally, the indirect effect through dietary diversity was also significant (ACME = −0.006, 95% CI: −0.010 to −0.003), suggesting that a partial mediation effect accounted for 18.79% of the total effect (Figure 2).

**Figure 2:**
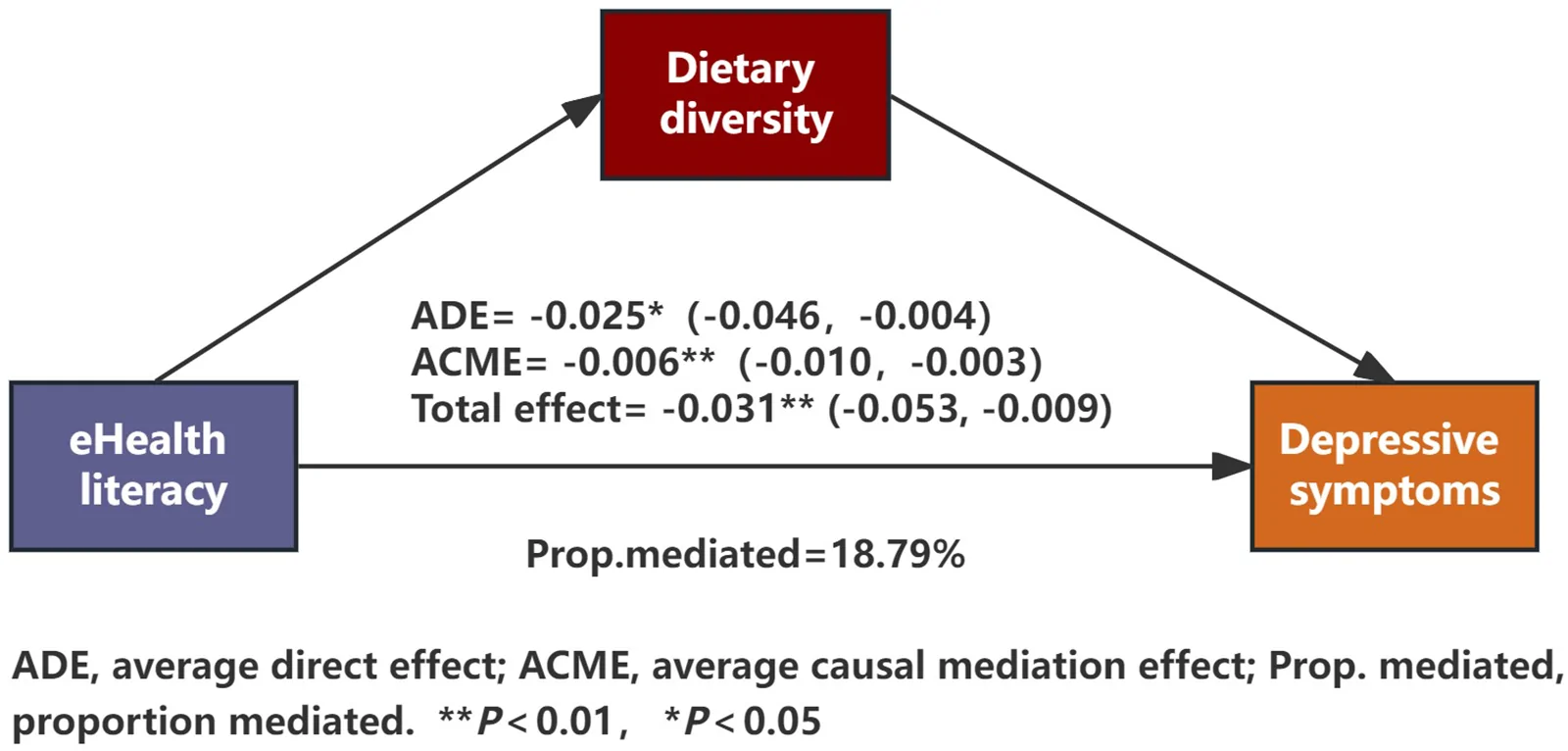
Analysis of the effects of dietary diversity on the relationship between eHealth literacy and depressive symptoms in patients with chronic diseases. The model was adjusted for age, sex, educational level, self-rated health status, occupation, alcohol consumption status, and sleep duration.

### 3.6 Multivariable logistic regression analysis of eHealth literacy and depressive symptoms

Table 3 presents the associations between eHealth literacy and depressive symptoms. In comparison to the high eHealth literacy group, the medium (OR = 1.34, 95% CI: 1.21 to 1.48) and low (OR = 1.64, 95% CI: 1.49 to 1.82) eHealth literacy groups exhibited significantly greater odds of depressive symptoms. Following further adjustment for dietary diversity, these ORs decreased to 1.28 (95% CI: 1.14 to 1.47) and 1.53 (95% CI: 1.38 to 1.72), respectively. This reduction suggests that dietary diversity partially accounts for the association between eHealth literacy and depressive symptoms, thereby supporting its potential role as a mediator. Model diagnostics revealed no significant multicollinearity among the independent variables (all VIF <2, mean VIF = 1.17), and the model exhibited good goodness-of-fit (Nagelkerke's R^2^ = 0.356).

**Table 3:** Associations of eHealth literacy with depressive symptoms in patients with chronic diseases.

eHealth literacy	N(total=2,655)	Odds ratio (95% CI)	
		Unadjusted for DDS	Adjusted for DDS
High eHealth literacy	914 (34.43%)	1 (Reference)	1 (Reference)
Medium eHealth literacy	516 (19.43%)	1.34 (1.21 to 1.48)**	1.28 (1.14 to 1.47)**
Low eHealth literacy	1225 (46.14%)	1.64 (1.49 to 1.82)*	1.53 (1.38 to 1.72)**

eHealth literacy was generated through latent profile analysis. All the models included age, sex, educational level, self-rated health status, occupation, alcohol consumption status, and sleep duration. **DDS**, dietary diversity score. The reduction in odds ratios between the unadjusted and adjusted models indicates the explanatory role of dietary diversity.

** *P* < 0.01.

* *P* < 0.05.

### 3.7 Interaction and joint analysis of dietary diversity and eHealth literacy with depressive symptoms

No significant multiplicative interaction was identified between eHealth literacy and dietary diversity concerning depressive symptoms in patients with chronic diseases; however, a significant additive interaction was observed (RERI = 0.65, 95% CI: 0.12 to 1.19; *P* = 0.017). A higher dietary diversity score correlated with reduced odds of depressive symptoms across all three eHealth literacy subgroups (Figure 3). Specifically, the odds ratios for individuals with a DDS of 7–9 compared with those with a DDS of 0–3 were 0.20 (95% CI: 0.09 to 0.46) for individuals with low eHealth literacy, 0.30 (95% CI: 0.11 to 0.75) for those with medium eHealth literacy, and 0.13 (95% CI: 0.07 to 0.40) for those with high eHealth literacy. These stratified associations are descriptive in nature. The presence of a statistically significant interaction was indicated by the significant additive interaction (RERI), while the multiplicative interaction did not reach significance.

**Figure 3:**
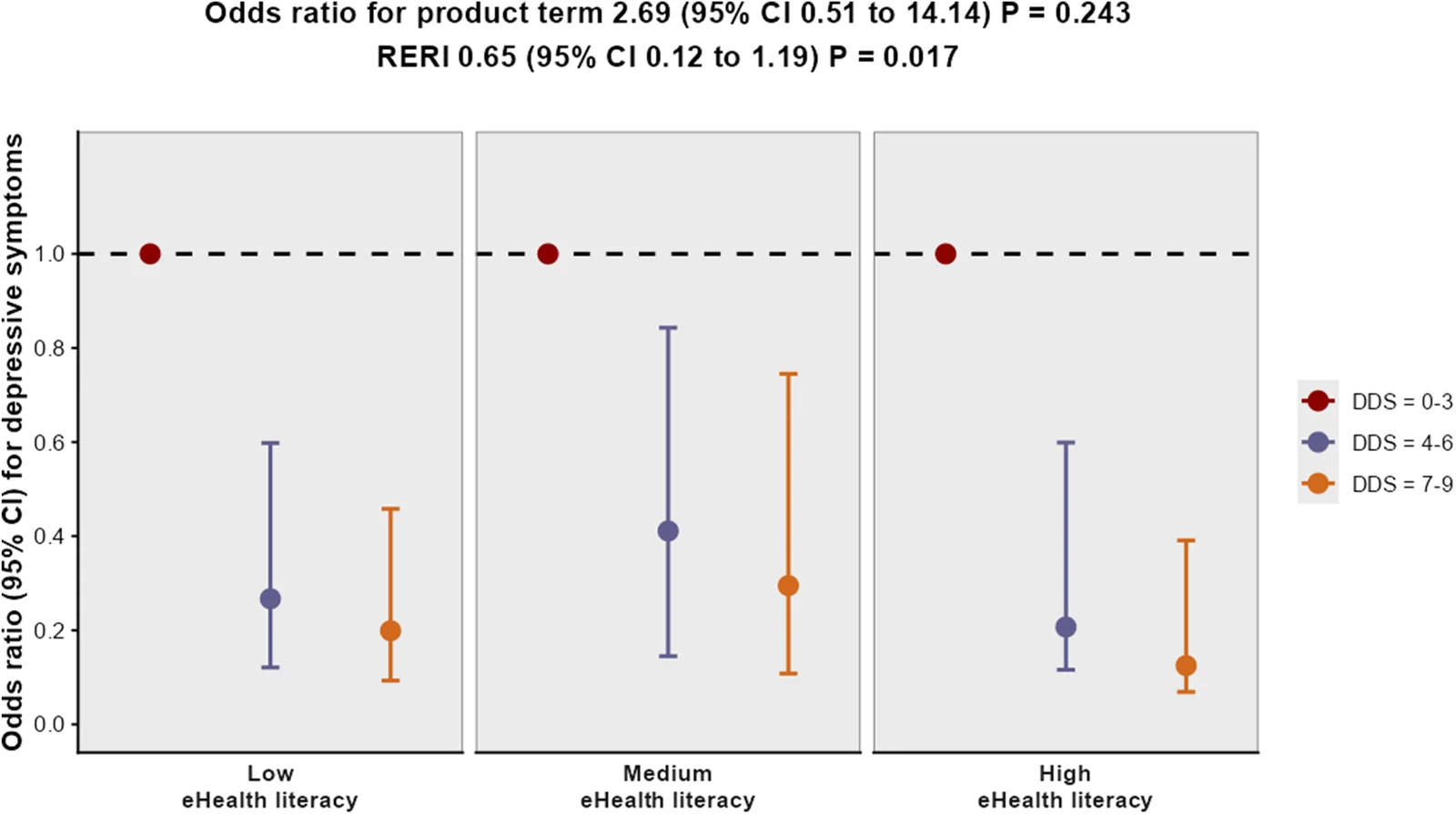
Associations of dietary diversity score with depressive symptoms according to eHealth literacy in patients with chronic diseases. **RERI**, relative excess risk due to interaction; **DDS,** dietary diversity score. Odds ratios were adjusted for age, sex, educational level, self-rated health status, occupation, alcohol consumption status, and sleep duration. Multiplicative interaction was evaluated using odds ratios for the product term between eHealth literacy (low v high) and dietary diversity score (0–3 v 7–9), and the multiplicative interaction was statistically significant when its confidence interval did not include 1. Additive interaction was evaluated using the RERI between eHealth literacy (low v high) and the dietary diversity score (0–3 v 7–9), and the additive interaction was statistically significant when its confidence interval did not include 0.

The joint associations between eHealth literacy and dietary diversity scores with depressive symptoms in patients with chronic diseases are shown in Figure 4. In comparison to those in the reference group (those with low eHealth literacy and DDS scores of 0–3), the odds of experiencing depressive symptoms progressively decreased with increasing eHealth literacy and higher dietary diversity scores. Notably, the OR for individuals who reported high eHealth literacy and a DDS ranging from 7 to 9 was 0.17 (95% CI: 0.05 to 0.56), indicating that eHealth literacy was most strongly protective. Similar dose‒response patterns were evident across the medium and high eHealth literacy subgroups.

**Figure 4:**
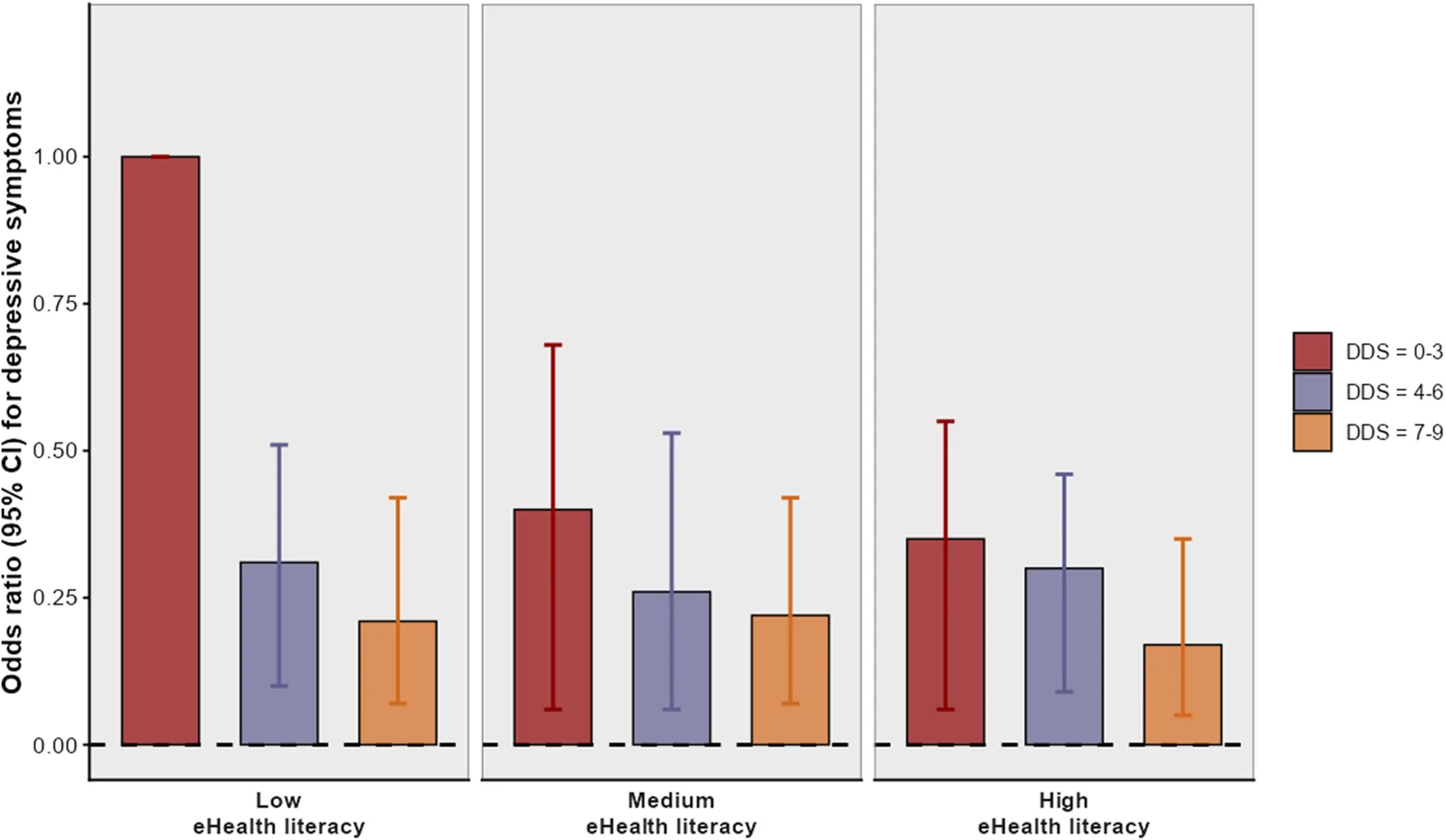
Joint associations of eHealth literacy and dietary diversity score with depressive symptoms in patients with chronic diseases. **DDS,** dietary diversity score. Odds ratios were adjusted for age, sex, educational level, self-rated health status, occupation, alcohol consumption status, and sleep duration.

### 3.8 Sensitivity analysis

To validate the robustness of our primary findings, we conducted two sets of sensitivity analyses. First, treating the PHQ-9 score as a continuous variable revealed that the significant associations and the mediating role of dietary diversity remained consistent with the primary analyses (Supplementary Table S5 and Supplementary Figure S2). Second, employing a stricter cutoff of ≥10 points resulted in directionally consistent associations that exhibited a stronger trend, particularly within the low eHealth literacy group (Supplementary Table S6 and Supplementary Figure S3). These results indicate that our main conclusions are robust across various analytical frameworks.

## 4 Discussion

The rapid advancement of the internet and information technology has rendered eHealth literacy a vital determinant of individual capability, directly affecting one's ability to access and utilize health information online and make informed health choices. Research has demonstrated that eHealth literacy significantly promotes individual health behaviors. Improving eHealth literacy enables the intentional acquisition of health knowledge, and individuals with elevated eHealth literacy are more inclined to adopt healthy behaviors (42). Using LPA, we identified distinct levels of eHealth literacy among patients with chronic diseases. Notably, participants with lower eHealth literacy demonstrated significantly greater odds of experiencing depressive symptoms and exhibited lower dietary diversity than those with higher eHealth literacy did. This association may stem from the fact that patients with low eHealth literacy often lack the skills necessary to effectively navigate digital health platforms. Consequently, they are less likely to access supportive health resources and health management information online, which may exacerbate feelings of helplessness and isolation, ultimately contributing to poor mental health outcomes (43). Furthermore, the weak linear correlations observed in our study indicate that simple linear relationships may not adequately capture the complex, multidimensional interactions among these factors. Therefore, it is essential to adopt more robust analytical approaches, such as LPA and mediation analysis, to elucidate the indirect and synergistic pathways underlying these associations. The strength of LPA in this study lies in its ability to identify naturally occurring subgroups without reliance on arbitrary cutoff scores. This data-driven approach enabled us to pinpoint specific vulnerable subgroups that might otherwise be obscured by a single total score or conventional cutoff. Such classification provides precise targets for tailored digital health interventions.

Dietary diversity has been identified as a significant mediator of the association between eHealth literacy and depressive symptoms. From a behavioral perspective, individuals with higher eHealth literacy are more inclined to seek, comprehend, and utilize health information to enhance their dietary practices. A cross-sectional study involving pregnant women revealed a significant correlation between eHealth literacy and nutritional self-efficacy, which acted as a mediator in the pathway to improved diet quality (44). The underlying mechanism of this indirect association may be that increased eHealth literacy allows individuals to navigate online nutritional information more effectively and adopt healthier eating patterns. Related research has indicated that eHealth literacy significantly promotes dietary diversity among rural people; through online platforms, individuals can access concepts of healthy food consumption and learn scientific methods for food pairing, thereby achieving a balanced diet and increasing diversity (45). Notably, consuming a variety of foods can assist patients with chronic diseases in developing positive and optimistic emotions, thereby increasing their life satisfaction (46, 47).

From a biological perspective, the significance of dietary diversity for mental health is well established. A varied diet ensures an adequate intake of essential nutrients, which is vital for maintaining mental well-being. A randomized controlled trial demonstrated that dietary interventions can effectively alleviate depressive symptoms (48). High dietary diversity is associated with increased intake of micronutrients, phytochemicals, and dietary fiber, which are correlated with a reduced risk of depression through multiple mechanisms, including antioxidant effects, anti-inflammatory responses, and regulation of the gut–brain axis (49). In contrast, a monotonous diet can result in deficiencies of specific nutrients, potentially leading to elevated homocysteine levels, impaired neurotransmitter synthesis, and gut microbiota dysbiosis, which may induce or exacerbate depressive symptoms (50–52). This relationship is further supported by a systematic review and meta-analysis indicating that higher dietary diversity is inversely associated with depression (27), as well as a study conducted in rural Western Kenya that revealed that greater dietary diversity correlated with lower depression scores, particularly among socioeconomically vulnerable individuals (53). Additionally, the gut–brain axis has emerged as a crucial pathway linking diet and mental health (54). A diverse diet promotes the growth of beneficial gut microbiota, which may regulate neurotransmitter synthesis and mitigate systemic inflammation, thereby potentially enhancing mood and alleviating depressive symptoms (55). This biological mechanism is especially important for patients with chronic diseases, who frequently experience chronic low-grade inflammation that may worsen both physical and mental health conditions.

The results of the present study revealed a significant additive interaction between eHealth literacy and dietary diversity, although no significant multiplicative interaction was detected. The notable RERI suggests that the combination of high eHealth literacy and high dietary diversity confers a level of protection against depressive symptoms that surpasses the sum of their individual effects. The absence of statistical significance for the product term does not imply that no additive or biological interaction exists between these two factors (56). The additive interaction model, which is grounded in the risk difference scale that quantifies absolute changes in risk, aligns more closely with the biological concept of synergy and is of greater relevance to public health (57). Previous research has established independent associations between health literacy, dietary behaviors, and mental health (58). Our findings contribute to this understanding by revealing a synergistic relationship, indicating that digital health empowerment and nutritional behaviors may mutually reinforce each other. This synergistic pattern is biologically plausible; individuals with higher eHealth literacy are better equipped to comprehend and apply complex dietary information, such as identifying nutrient-dense foods and recognizing balanced meal structures. Furthermore, they are more adept at critically evaluating conflicting online nutritional advice. This dual capability may enhance the mental health benefits associated with diverse diets, thereby fostering a positive feedback loop between digital skills and healthy eating. Previous meta-analyses have consistently demonstrated that dietary diversity is inversely associated with depression (59), whereas higher eHealth literacy is correlated with improved health self-management (43).

From a public health perspective, this notable additive interaction has significant implications for high-risk rural patients. The synergistic protective association suggests that the combined benefits of high eHealth literacy and high dietary diversity surpass the sum of their individual advantages—a “1 + 1 > 2” effect. These findings indicate that isolated interventions aimed at enhancing digital skills or nutrition alone may be inadequate in resource-limited settings. Consequently, our results support the implementation of integrated strategies that combine digital health literacy training with nutritional counseling. For instance, community-based programs could offer nutritional education via mobile health applications while concurrently training patients to assess the credibility of health information sources. Furthermore, customized digital platforms that deliver straightforward, actionable dietary guidance may prove more effective when combined with ongoing digital skills training. Such comprehensive approaches may better alleviate the burden of depressive symptoms in vulnerable populations. Future public health initiatives should prioritize patients who exhibit both low eHealth literacy and poor dietary habits.

These findings hold significant implications for the management of chronic diseases. In both clinical and public health settings, a comprehensive approach that integrates digital health literacy training with nutritional counseling may prove more effective than individual interventions. Efforts to enhance eHealth literacy can not only alleviate depressive symptoms directly but also provide indirect benefits by fostering dietary diversity. Given the higher prevalence of low eHealth literacy among rural populations with limited medical resources, targeted digital health education programs that incorporate nutritional guidance could be especially advantageous for these vulnerable groups. Individuals with low eHealth literacy frequently encounter difficulties in acquiring, understanding, and applying online health information; as a result, they struggle to obtain appropriate dietary advice to enhance their diet quality and often exhibit poor self-management of health. Consequently, patients with low eHealth literacy and limited dietary diversity represent a high-risk group for the development of depressive symptoms and warrant focused attention.

The findings of this study indicate several avenues for future research and practice. First, considering the observed associations among eHealth literacy, dietary diversity, and depressive symptoms, subsequent studies could investigate whether targeted interventions aimed at enhancing eHealth literacy might lead to improved dietary behaviors and mental health outcomes among rural residents with chronic diseases. Second, additional research is necessary to assess how digital health platforms and community-based health education programs could facilitate the adoption of diverse dietary patterns within this population. Such initiatives may elucidate the potential pathways through which eHealth literacy impacts dietary quality and, subsequently, depressive symptoms. Nevertheless, the causal mechanisms underlying these relationships have yet to be established, necessitating longitudinal or interventional studies to validate these hypotheses.

## 5 Limitations and implications

This study has several limitations that suggest avenues for future research. First, owing to its cross-sectional design, it is not possible to establish causal relationships among eHealth literacy, dietary diversity, and depressive symptoms, and the potential for reverse causation cannot be ruled out (e.g., depressive symptoms may contribute to poorer dietary habits or diminished eHealth literacy). Future longitudinal or interventional studies are necessary to confirm the temporal sequence of these associations. Second, the assessment instruments employed possess inherent limitations. The PHQ-9 was utilized to identify probable depressive disorder using a standard cutoff score of ≥5; however, as a self-reported scale, it may not accurately represent a formal clinical diagnosis of depression. The DDS was derived from a three-day dietary recall, capturing the variety of food groups consumed, yet it lacks detailed information on portion sizes and consumption frequency, thereby preventing the estimation of total caloric or specific nutrient intake. Furthermore, eHEALS assesses subjective confidence rather than objective digital skills. These self-reported measures are vulnerable to recall bias and social desirability bias; although we excluded patients with severe psychiatric or memory disorders, residual bias may still persist. Third, the profiles identified through LPA are contingent upon the model and data; the number and characteristics of the subgroups may differ when they are applied to alternative samples or model specifications. Fourth, eHealth literacy and dietary diversity are dynamic factors that may evolve over time; future research could investigate the impact of their trajectories on health outcomes. Fifth, this study was conducted exclusively within the rural population of Ningxia, China, which may limit the generalizability of our findings to other regions. Sixth, the use of complete-case analysis, which excludes participants with missing data, along with the exclusion of individuals with severe illnesses, may introduce selection bias that could further affect the generalizability of our results. Finally, residual confounding remains a significant concern in observational studies. Variables such as physical activity, body mass index (BMI), medication use, disease severity, and specific types of chronic diseases may independently influence both dietary diversity and depressive symptoms, potentially confounding the observed associations. For example, patients with severe chronic conditions may experience both restricted dietary intake and heightened psychological distress. Therefore, future studies should incorporate comprehensive clinical data, including objective measures of physical activity, BMI, polypharmacy, and disease-specific severity scores, to further mitigate residual confounding.

Despite these limitations, this study has several important clinical and public health implications. First, through LPA, we identified distinct profiles of eHealth literacy among rural patients with chronic diseases and comprehensively assessed their associations with depressive symptoms. The results of the mediation analysis provided empirical support for the potential mediating role of dietary diversity, which accounted for 18.79% of the total effect. Given the observational nature of this study, these findings should be viewed as hypothesis-generating. Therefore, rather than assessing causality, our results indicate that future interventions aimed at enhancing eHealth literacy and dietary diversity may be promising for improving mental health outcomes. Second, the significant additive interaction suggests a synergistic effect, indicating that patients with both high eHealth literacy and high dietary diversity exhibited the lowest odds of depressive symptoms. These findings establish a theoretical foundation for the development of integrated prevention strategies that combine digital health literacy training with nutritional counseling, particularly in rural areas with limited medical resources. Finally, this study presents low-cost, feasible intervention ideas for grassroots public health, serving as a reference for tailoring health education programs for chronic disease patients in resource-limited settings.

## 6 Conclusion

Among patients with chronic diseases in rural areas, low eHealth literacy was linked to an increased likelihood of depressive symptoms, with dietary diversity potentially serving as a partial mediator of this relationship. Furthermore, a significant additive interaction was identified, indicating a synergistic protective effect; patients who reported both high eHealth literacy and high dietary diversity demonstrated the lowest likelihood of depressive symptoms. These findings imply that enhancing dietary patterns may be beneficial for improving mental health outcomes, particularly for individuals with low eHealth literacy. Consequently, comprehensive interventions aimed at addressing mental health in rural chronic disease patients should consider incorporating strategies to enhance eHealth literacy alongside nutritional support. Future longitudinal studies and randomized controlled trials are necessary to validate these associations and further assess the potential advantages of interventions focused on eHealth literacy and dietary diversity.

## Data Availability

The original contributions presented in the study are included in the article/Supplementary Material, further inquiries can be directed to the corresponding author.
