# Supplementary material for: Associations of eHealth literacy and dietary diversity with depressive symptoms among patients with chronic diseases in rural Western China
Source: Front Digit Health. 2026 Sep 25;8:1920279. doi: 10.3389/fdgth.2026.1920279 (PMC13649753; doi:10.3389/fdgth.2026.1920279)
Supplement: Supplementary file 1 [file Datasheet1.pdf]

Supplementary Materials

Table S1 Distribution of participant characteristics using ≥5 and ≥10 cut-offs of the PHQ-9.

Table S2. Univariate analysis of potential covariates associated with depressive symptoms.

Table S3. Fitting index of latent profile model for eHealth literacy

Figure S1. Profile plot of the three-class latent profile model for eHealth literacy.

Table S4. Comparison of baseline characteristics between excluded and included participants.

Table S5. Sensitivity analysis of the association between eHealth literacy and depressive symptoms using the continuous PHQ-9 score.

Figure S2. Sensitivity analysis of the mediation model using the continuous PHQ-9 score.

Table S6. Sensitivity analysis of the association between eHealth literacy and depressive symptoms using a stricter cut-off of ≥10 points on the PHQ-9.

Figure S3. Sensitivity analysis of the mediation model using a stricter cut-off of ≥10 points for the PHQ-9.

TABLE S1 Distribution of participant characteristics using ≥5 and ≥10 cut-offs of the PHQ-9.

Characteristics	Cut-off ≥5		Cut-off ≥10	
	**Non-significant depressive symptoms group (1702, 64.11%)**	**Depressive symptoms group (953, 35.89%)**	**Non-significant depressive symptoms group (2364, 89.04%)**	**Depressive symptoms group (291, 10.96%)**
**Age(years), mean ± SD**	62.16±11.04	64.61±10.83	62.84±10.98	64.69±11.03
**Gender, n (%)**				
Men	1042(61.22)	496(52.05)	1398(59.14)	140(48.11)
Women	660(38.78)	457(47.95)	966(40.86)	151(51.89)
**Marital status, n (%)**				
Married	1672(98.24)	935(98.11)	2319(98.10)	288(98.97)
Unmarried	30(1.76)	18(1.89)	45(1.90)	3(1.03)
**Educational level, n (%)**				
Uneducated	638(37.49)	478(50.16)	931(39.38)	185(63.57)
Primary school	651(38.25)	335(35.15)	915(38.71)	71(24.40)
Junior high school and above	413(24.27)	148(14.69)	518(21.91)	35(12.03)
**Occupation, n (%)**				
Agricultural work	1294(76.03)	677(71.04)	1775(75.08)	196(67.35)
Non-Agricultural work	408(23.97)	276(28.96)	589(24.89)	95(32.65)
**Self-rated health status, n (%)**				
Very good	38(2.23)	6(0.63)	42(1.78)	2(0.69)
Good	249(14.63)	39(4.09)	284(12.01)	4(1.37)
Fair	521(30.61)	124(13.01)	622(26.31)	23(7.90)
Poor	659(38.72)	414(43.44)	975(41.24)	98(33.68)
Very poor	235(13.81)	370(38.82)	441(18.65)	164(56.36)
**Dietary diversity, mean ± SD**	6.94±1.41	6.52±1.59	6.88±1.45	6.06±1.64
**Smoking status, n (%)**				
Current smoke	400(23.50)	177(18.57)	525(22.21)	52(17.87)
Never smoker	1302(76.50)	776(81.43)	1839(77.79)	239(82.13)
**Alcohol consumption status, n (%)**				
Current drinker	139(8.17)	44(4.62)	171(7.23)	12(4.12)

Never drinker	1563(91.83)	909(95.38)	2193(92.77)	279(95.88)
**Sleep duration, mean ± SD**	6.95±1.41	6.23±1.77	6.80±1.49	5.81±2.03
**Exercise frequency per week, n (%)**				
0 times	774(45.48)	582(50.58)	1088(46.02)	168(57.73)
1-2 times	207(12.16)	105(11.02)	289(12.23)	23(7.9)
3-5 times	290(17.04)	118(12.38)	381(16.12)	27(9.28)
≥6 times	431(25.32)	248(26.02)	606(25.63)	73(25.09)
**Annual income, M (IQR)**	10790.50(6790.50,17430.25)	10020.2(6170.00,17620.50)	10525.33(6680.24,17250.75)	10775.50(6440.1,17494.43)

Note: PHQ-9, Patient Health Questionnaire-9. **SD**, standard deviation; **IQR**, interquartile range. The cut-off of ≥5 indicates mild depressive symptoms, while the cut-off of ≥10 indicates moderate-to-severe depressive symptoms. All values were derived from the complete-case dataset (n = 2,655).

TABLE S2. Univariate analysis of potential covariates associated with depressive symptoms.

Characteristics	OR (95% CI)	*P*
**Age (years)**	1.02 (1.01 to 1.03)	< 0.001
**Gender**		
Men	1 (Reference)	-
Women	1.45 (1.24 to 1.71)	< 0.001
**Marital status,**		
Married	1 (Reference)	-
Unmarried	0.93 (0.52 to 1.68)	0.815
**Educational level,**		
Uneducated	1 (Reference)	-
Primary school	0.69 (0.58 to 0.82)	< 0.001
Junior high school and above	0.45 (0.36 to 0.57)	< 0.001
**Occupation,**		
Agricultural work	1 (Reference)	-
Non-Agricultural work	1.29 (1.08 to 1.55)	0.005
**Self-rated health status**	2.29 (2.08 to 2.53)	< 0.001
**Smoking status**		
Never smoker	1 (Reference)	-
Current smoke	1.25 (0.91 to 1.71)	0.174
**Alcohol consumption status,**		
Never drinker	1 (Reference)	-
Current drinker	1.84 (1.30 to 2.60)	0.001
**Sleep duration**	0.75 (0.71 to 0.79)	< 0.001
**Exercise frequency per week**	1.04 (0.99 to 1.09)	0.076
**Annual income**	0.99 (0.99 TO 1.01)	0.488

TABLE S3. Fitting index of latent profile model for eHealth literacy

Model	AIC	BIC	aBIC	Entropy	*P*		Category probability (%)
					BLRT	VLMR	
1c	24632.56	24667.86	24648.80				
2c	16615.68	16674.51	16642.74	0.975	0.01	0.01	64.07/35.93
3c	14255.55	14337.93	14293.45	0.979	0.01	0.01	46.14/19.43/34.43
4c	8318.92	8424.83	8367.64	1	0.1077	0.1134	47.38/13.02/18.15/21.35
5c	7852.25	7981.70	7911.80	0.998	0.1594	0.1628	16.98/47.34/1.21/21.36/13.11

Note: AIC, Akaike Information Criterion; **BIC**, Bayesian Information Criterion; **aBIC**, sample-size adjusted BIC; **BLRT**, Bootstrap Likelihood Ratio Test; **VLMR**, Vuong-Lo-Mendell-Rubin test.

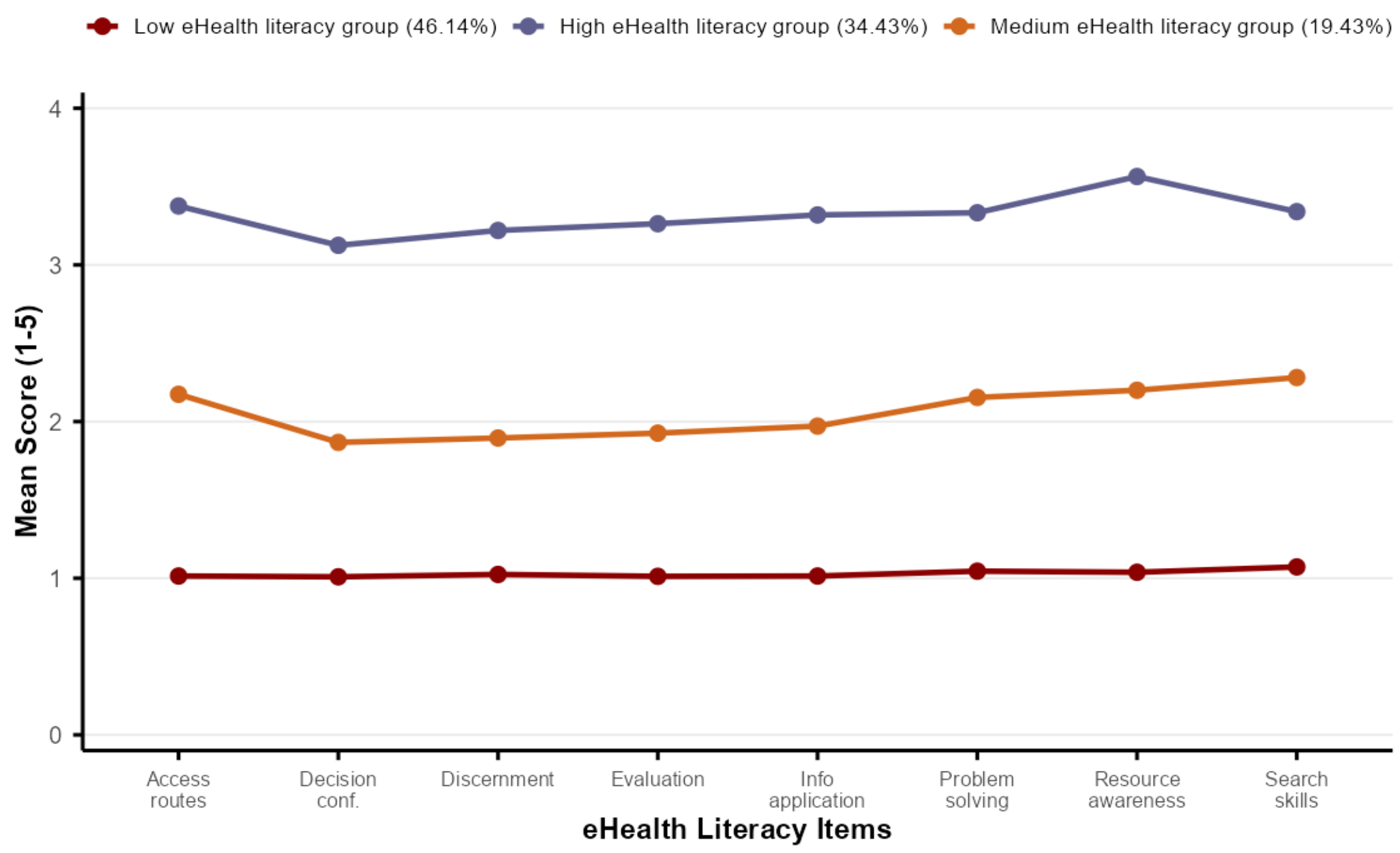

FIGURE S1. Profile plot of the three-class latent profile model for eHealth literacy.

TABLE S4 Comparison of baseline characteristics between excluded and included participants.

Characteristics	Excluded participants (n = 3,819)	Included participants (n = 2,655)	*P*
**Age(years), mean ± SD**	62.36±11.14	63.04±11.03	0.062
**Gender, n (%)**			< 0.001
Men	1347(35.26)	1538(57.93)	
Women	2472(64.74)	1117(42.07)	
**Marital status, n (%)**			0.132
Married	3728(97.63)	2607(98.19)	
Unmarried	91(2.37)	48(1.81)	
**Educational level, n (%)**			0.096
Uneducated	1644(43.05)	1116(42.03)	
Primary school	1430(37.45)	986(37.14)	
Junior high school and above	745(19.50)	553(20.83)	
**Occupation, n (%)**			0.083
Agricultural work	2758(72.21)	1971(74.24)	
Non-Agricultural work	1061(27.79)	684(25.76)	
**Self-rated health status, n (%)**			0.150
Very good	49(1.28)	44(1.66)	
Good	369(9.65)	288(10.85)	
Fair	897(23.49)	645(24.29)	
Poor	1602(41.96)	1073(40.41)	
Very poor	902(23.62)	605(22.79)	
**Depressive symptoms, n (%)**			0.083
Yes	1308(34.25)	953(35.89)	
No	2511(65.75)	1702(64.11)	
**Dietary diversity, mean ± SD**	6.52±1.36	6.79±1.49	0.066
**Smoking status, n (%)**			< 0.001
Current smoke	450(11.78)	577(21.73)	
Never smoker	3369(88.22)	2078(78.27)	
**Alcohol consumption status, n (%)**			< 0.001
Current drinker	146(3.83)	183(6.89)	
Never drinker	3673(96.17)	2472(93.11)	
**Sleep duration, mean ± SD**	6.98±1.40	6.69±1.58	0.897
**Exercise frequency per week, n (%)**			0.127
0 times	1864(48.81)	1256(47.31)	
1-2 times	388(10.17)	312(11.75)	
3-5 times	563(14.74)	408(15.37)	

≥6 times	1004(26.28)	679(25.57)	
**Annual income, M (IQR)**	30565.00(16942.00,55426.00)	31271.00(17460.00,56801.00)	0.085

TABLE S5 Sensitivity analysis of the association between eHealth literacy and depressive symptoms using the continuous PHQ-9 score.

eHealth literacy	N(total=2,655)	*β* (95% CI)	
		Unadjusted for DDS	Adjusted for DDS
High eHealth literacy	914 (34.43%)	0 (Reference)	0 (Reference)
Medium eHealth literacy	516 (19.43%)	0.24(0.11 to 0.52) *	0.19(0.07 to 0.47) *
Low eHealth literacy	1225 (46.14%)	0.64(0.25 to 1.02) **	0.52(0.14 to 0.91) *

Note: eHealth literacy was generated through latent profile analysis. All the models included age, sex, educational level, self-rated health status, occupation, alcohol consumption status, and sleep duration. **DDS**, dietary diversity score. The reduction in odds ratios between the unadjusted and adjusted models indicates the explanatory role of dietary diversity. $^{**}P$<0.01, $^{*}P$<0.05

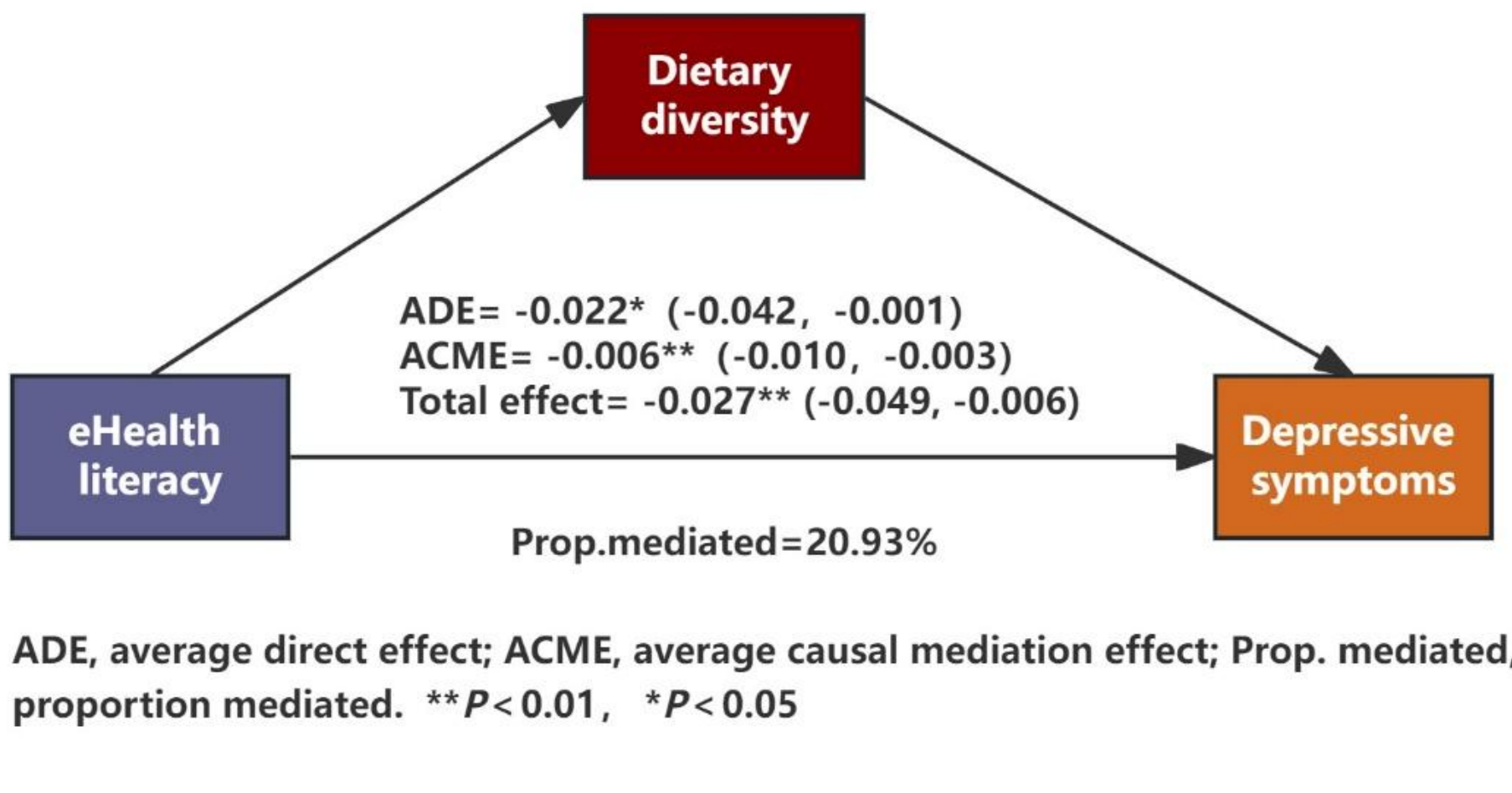

FIGURE 2. Sensitivity analysis of the mediation model using the continuous PHQ-9 score.

Note: The model was adjusted for age, sex, educational level, self-rated health status, occupation, alcohol consumption status, and sleep duration.

TABLE S6 Sensitivity analysis of the association between eHealth literacy and depressive symptoms using a stricter cut-off of ≥10 points on the PHQ-9.

eHealth literacy	N(total=2,655)	Odds ratio (95% CI)	
		Unadjusted for DDS	Adjusted for DDS
High eHealth literacy	914 (34.43%)	1 (Reference)	1 (Reference)
Medium eHealth literacy	516 (19.43%)	1.51(0.94 to 2.32) **	1.45(0.87 to 2.13) **
Low eHealth literacy	1225 (46.14%)	1.86(1.09 to 2.69) *	1.77(1.01 to 2.53) *

Note: eHealth literacy was generated through latent profile analysis. All the models included age, sex, educational level, self-rated health status, occupation, alcohol consumption status, and sleep duration. **DDS**, dietary diversity score. The reduction in odds ratios between the unadjusted and adjusted models indicates the explanatory role of dietary diversity. $^{**}P<0.01$, $^{*}P<0.05$

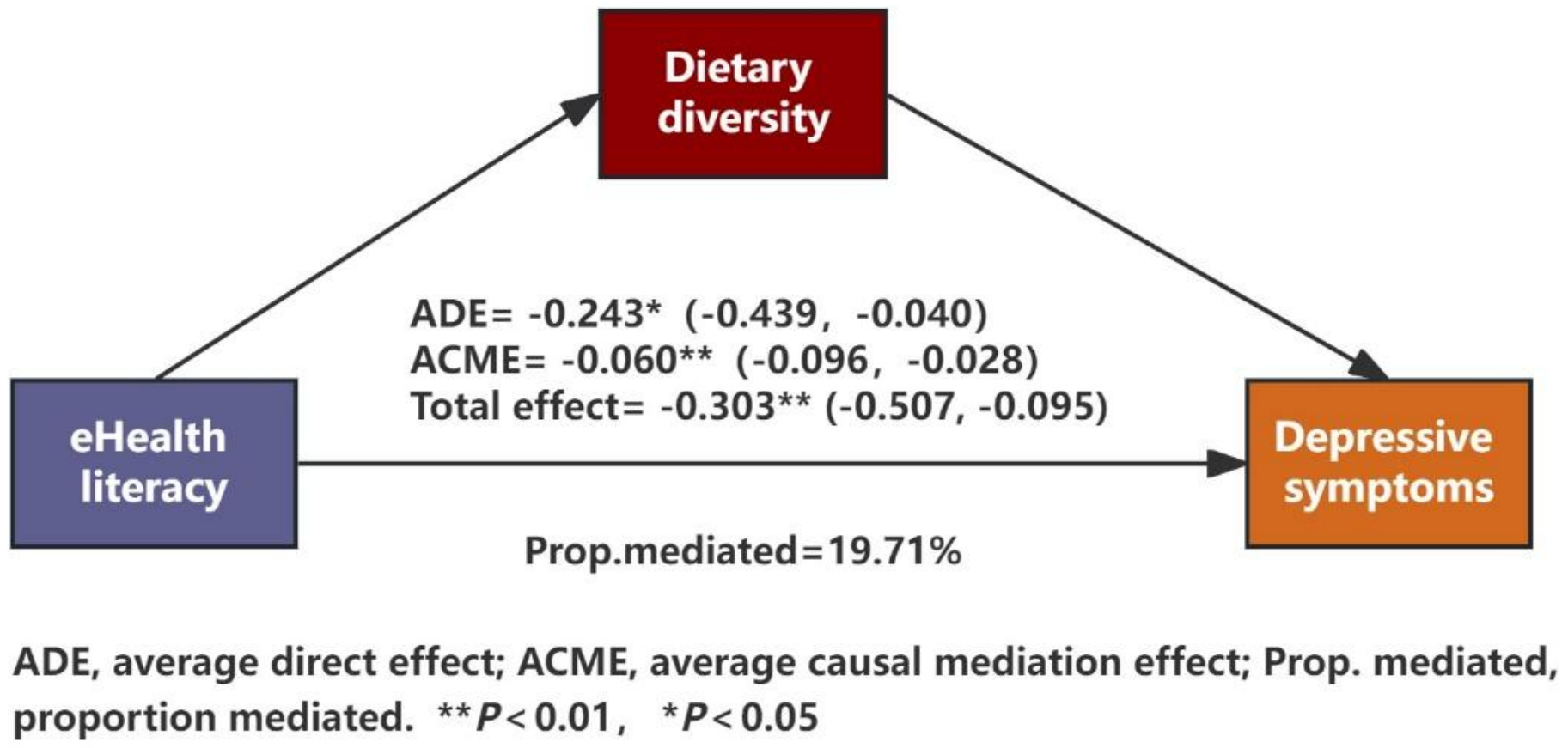

FIGURE S3. Sensitivity analysis of the mediation model using a stricter cut-off of ≥10 points for the PHQ-9.

Note: The model was adjusted for age, sex, educational level, self-rated health status, occupation, alcohol consumption status, and sleep duration.
